# Role of subjective snoring in dementia risk

**DOI:** 10.1093/sleep/zsae162

**Published:** 2024-07-15

**Authors:** Naomi L Gaggi, Donald L Bliwise, Andrew W Varga

**Affiliations:** Department of Psychiatry, NYU Grossman School of Medicine, New York, NY, USA; Department of Neurology, Sleep Center, Emory University School of Medicine, Atlanta, GA, USA; Mount Sinai Integrative Sleep Center, Division of Pulmonary, Critical Care, and Sleep Medicine, and Friedman Brain Institute, Icahn School of Medicine at Mount Sinai, New York, NY, USA

Cognitive decline and poor sleep health are suggested to be interconnected; not only is disrupted sleep suggested to predict cognitive decline [1, 2], but evidence also suggests that there are various pathways linking sleep and Alzheimer’s disease (AD) pathology [3, 4]. One possible mechanism linking sleep pathology and incipient dementia is obstructive sleep apnea (OSA). OSA has been associated with aging, higher body mass index (BMI), and cardiovascular disease [5, 6]. In the absence of objective measurement of OSA, snoring is often used as an imperfect proxy for the condition. For example, emerging evidence suggests that individuals with OSA and those who demonstrate sleep-disordered breathing, including individuals who snore, have a higher incidence of cognitive decline [7].

Gao et al. [8] aimed to investigate the relationship between snoring and dementia, with respect to BMI, age, and genetic predispositions in a sample of 451,250 individuals from the UK Biobank [9] using causal inference statistical analyses. The high prevalence of snoring in individuals with OSA may suggest that snoring can be a potential indicator of subsequent cognitive decline; however, more research is needed to understand this potential association. Some studies, with mostly small samples, have examined the relationship between cognition and snoring with mixed and even null results [10–13]. Gao et al. [8] addressed the relationship between snoring and dementia by using a large dataset and stratified subgroups of dementia (i.e. all-cause dementia, AD, and vascular dementia), which allowed potentially further mechanistic insights into how OSA may impact subsequent cognitive decline.

Perhaps somewhat surprisingly, Gao et al. [8] found an association between self-reported snoring and a ***lower*** risk of all-cause dementia and AD. Given at least some (albeit imperfect) associations between self-reported snoring and OSA, these results initially appear paradoxical, as their opposite would have been predicted (i.e. snoring positively associated with incident dementia). However, because BMI was also noted to be negatively associated with risk for incident dementia (i.e. lower BMI with higher risk for later dementia), the authors interpreted their negative association between snoring and subsequent dementia somewhat differently. More specifically, they viewed their data as indicative of reverse causation, i.e. that lower BMI and inferred weight loss were suggestive of the frailty often accompanies the prodromal phase of dementia. Interestingly, because OSA has been associated with frailty and weight loss in very old age [14, 15], the predicted relationship between OSA and impaired cognition [16, 17] might still be compatible with these findings, if one assumes that weight loss in old age is simultaneously both a proxy for the development of OSA and a consequence of incipient dementia.

An important alternative interpretation of the finding may have to do with the nature of the UK Biobank question regarding snoring, which was, “does your partner or a close relative or friend complain about your snoring?” Individual snorers who live alone are thus likely to answer “no” to this question but may also represent a subset with a higher risk for dementia due to isolation and reduced support for activities of daily living. Controlling for the variable “household size” did not strongly influence the associations, but this remains a caveat to consider.

One way to check whether OSA might mediate the observed associations with incident dementia would be to examine the impact of OSA diagnosis on the findings. Unfortunately, this effort becomes largely aspirational within this data set. The reported prevalence of OSA in the UK Biobank (< 1%) (Table 1 in Gao et al.) is vastly lower than one would expect based on other population-based studies of OSA, which consequently severely limits the power for examining whether the negative association between snoring and incident dementia was modified by OSA. The authors report that in the much larger subpopulation (99+ %) without known OSA, the inverse association between snoring and incident dementia remains intact, but that is largely tautological, given the size of that subpopulation relative to the total population. In short, the stratified analyses based on the small number of identified OSA cases do not help much in our understanding of the provocative phenomenon observed here.

Despite these counterintuitive findings, the UK Biobank represents an invaluable resource for sleep-related research. The size of the population allows for genotyping on a massive scale. Although the lack of assessment of OSA is a frank weakness, self-reported snoring in this data set indeed behaves in a predictable manner, to the extent that it increased with age, was more pronounced in men and was correlated with various cardiometabolic conditions. Similarly, the association between the APOE4 genotype and the development of dementia in the Biobank is confirmatory of dozens of studies demonstrating the risk of such genetic predisposition in much smaller samples. The ultimate meaning of the relationship between lower rates of snoring and higher rates of incident dementia reported here will await future studies with more accurate assessments of both snoring and OSA. The current results, however, do raise the equally intriguing but stark possibility that the association between OSA and the development of dementia in old age is essentially null.
